# School-based high-intensity interval training for children: a focused systematic review of implementation and school-relevant outcomes

**DOI:** 10.3389/fped.2026.1952928

**Published:** 2026-09-28

**Authors:** Lizhuo Yang, Liping Lin, Zhuomei Lan

**Affiliations:** 1 Wuhan Sports University, Wuhan, Hubei, China; 2 Guandian Middle School, Mingguang, Anhui, China; 3 Guandian Hospital, Mingguang, Anhui, China

**Keywords:** children, high-intensity interval training, narrative synthesis, physical education, school-based intervention, systematic review

## Abstract

**Background:**

Schools are an important setting for physical-activity promotion, but limited curriculum time can constrain the implementation of exercise programmes. High-intensity interval training (HIIT) may offer a brief format for increasing vigorous activity during physical education (PE), classroom activity breaks, or other school-day opportunities. This review examined school-based HIIT delivery models, physical and cardiovascular outcomes, cognition and other school-relevant outcomes, and implementation, feasibility, and safety reporting.

**Methods:**

Five electronic databases were searched for English-language studies published from January 2010 to May 9, 2026. Two reviewers independently screened records, assessed full texts, extracted data, and appraised risk of bias, with disagreements resolved by consensus. Eligible controlled studies evaluated a clearly defined HIIT intervention delivered in a school setting to children aged approximately 5–12 years and reported at least one physical, cardiovascular, cognitive, school-relevant, or implementation outcome. Owing to substantial heterogeneity in interventions and outcomes, findings were synthesised narratively in accordance with the Synthesis Without Meta-analysis (SWiM) reporting guideline.

**Results:**

The searches identified 3,508 records; 1,370 duplicates were removed, 2,138 records were screened, 94 full-text reports were assessed, and six studies were included. Analysed sample sizes ranged from 18 to 67 participants. Interventions ranged from 6-minute classroom micro-sessions to 20-minute PE-based programmes delivered for 4–12 weeks. Some studies reported between-group or group-by-time findings favouring HIIT for selected functional-strength, aerobic-fitness, arterial-stiffness, and heart-rate-variability outcomes; other findings were limited to within-group change, and effects were not consistent across protocols. One study assessed inhibitory control and reported improvements after both HIIT and moderate-intensity continuous training, providing no evidence of a HIIT-specific cognitive advantage. No included study directly assessed mental health, academic attainment, attendance, or classroom engagement. Three studies were judged to have a high overall risk of bias and three raised some concerns.

**Conclusions:**

School-based HIIT may be a brief adjunct to, rather than a replacement for, comprehensive PE. Preliminary findings suggest possible benefits for selected fitness and cardiovascular outcomes, while evidence for cognitive, mental-health, and academic benefits remains limited or absent. Given the small evidence base and risk-of-bias concerns, these findings should be interpreted cautiously. The current findings apply to the primary-school-aged samples represented and should not be extrapolated to adolescents. Larger, adequately powered school trials with stronger implementation and outcome reporting are required.

## 1 Introduction

Schools reach most children and adolescents during a period in which physical-activity habits, health behaviours, social participation, and learning routines are being established. The World Health Organization recommends that school-aged children and adolescents accumulate an average of at least 60 min per day of moderate-to-vigorous physical activity, yet most adolescents do not meet this recommendation (1–3). Schools are therefore frequently asked to contribute to health promotion, but feasibility is constrained by curriculum pressure, limited equipment, staff workload, and the need to protect learning time. The practical question is not simply whether exercise can improve a physiological outcome under controlled conditions, but whether a school can deliver an intervention repeatedly, safely, and acceptably within an ordinary timetable.

High-intensity interval training (HIIT) comprises repeated brief bouts of vigorous or near-maximal activity separated by planned recovery periods. HIIT can be adapted to running, games, body-weight circuits, rope skipping, or skill-based activities, making it a plausible school-day strategy when the aim is to increase vigorous activity without requiring a separate lesson or specialised equipment. However, the term encompasses interventions that differ markedly in exercise mode, work-to-rest ratio, session duration, intensity monitoring, delivery agent, and comparator. A 6-minute teacher-led classroom circuit and a 20-minute PE-based multi-activity programme should therefore not be treated as interchangeable interventions (4–6). Pediatric reviews use differing intensity thresholds (19). A youth expert statement characterises high-intensity interval exercise as repeated intense bouts performed at approximately 85%–95% of maximal heart rate or 80%–100% of peak work rate, separated by rest or lower-intensity recovery (20).

The evidence base has expanded rapidly. Duncombe et al. (7) identified 42 school-based HIIT interventions and reported improvements in several health outcomes, while emphasising substantial heterogeneity and frequent risk-of-bias concerns. Liu et al. (8) subsequently identified 45 interventions and found that process-evaluation reporting was commonly incomplete. Although these reviews demonstrate growing interest in school-based HIIT, relatively limited attention has been paid to how HIIT programmes are implemented and adapted within routine school environments and whether such adaptations support feasible delivery. Schools provide structured opportunities for exercise delivery, particularly through physical education and classroom-based activity sessions, but they also present substantial contextual variability. Differences in student characteristics, class size, lesson structure, available facilities, teacher experience, and curricular demands may require HIIT protocols to be modified to suit local conditions. However, how school-based HIIT interventions are adapted, what implementation challenges arise, and whether these adaptations influence feasibility, fidelity, acceptability, or sustainability remain insufficiently synthesised. These reviews establish that school-based HIIT is not an uncharted topic. A focused review must therefore distinguish the outcomes directly measured in individual trials from the broader health, wellbeing, and educational claims sometimes used to justify implementation.

The present review applies a school-relevance lens to eligible controlled studies. It maps delivery characteristics and examines whether evidence on physical fitness and cardiovascular function is accompanied by evidence on cognition, mental health, academic performance, attendance, classroom engagement, safety, acceptability, and implementation fidelity. This distinction is important because a plausible mechanism or a general association between physical activity and wellbeing does not demonstrate that a specific school-based HIIT programme improves a school-relevant outcome.

The review addressed four questions: (1) Which school-based HIIT delivery models have been evaluated in children? (2) What effects have been reported across physical-fitness, cardiovascular, cognitive, mental-health, academic, and implementation outcomes? (3) How completely are intervention dose, fidelity, safety, and acceptability reported? and (4) What methodological limitations and evidence gaps should guide future school-based trials?

## 2 Methods

### 2.1 Protocol, reporting framework, and review design

This focused systematic review with narrative synthesis was prepared in accordance with the Preferred Reporting Items for Systematic Reviews and Meta-Analyses (PRISMA) 2020 statement and the PRISMA-S extension for search reporting (9, 10). The review team reports that the review question, eligibility criteria, information sources, screening procedures, data items, risk-of-bias approach, and synthesis plan were specified during conduct of the review, but this timing cannot be independently verified from a prospective record. The outcome domains used for data extraction and synthesis were defined before data extraction began. The review was not prospectively registered, and no standalone prospective protocol was prepared. After completion, it was retrospectively registered on the Open Science Framework on 24 August 2026 (registration DOI: 10.17605/OSF.IO/E95N3).

### 2.2 Eligibility criteria

Eligibility criteria were structured using the Population, Intervention, Comparator, Outcomes, and Study design (PICOS) framework. Eligible participants were children aged approximately 5–12 years who were recruited through primary or secondary schools. Eligible interventions were clearly defined HIIT programmes delivered during the school day or embedded in school-related provision, including PE, classroom activity breaks, recess, or before- and after-school sessions administered by school or research staff. The intervention was required to include repeated high-intensity work bouts separated by planned recovery or lower-intensity periods. Published pediatric definitions use differing numerical intensity thresholds (19). Because the original eligibility criterion did not specify one numerical cutoff, the revised synthesis does not retrospectively recast the youth guidance values as prespecified eligibility thresholds. Instead, the high-intensity component was identified from information reported in the primary study regarding the prescribed or monitored exercise intensity, such as heart rate, rating of perceived exertion, external work rate or speed, vigorous-intensity monitoring, or an explicit maximal or near-maximal exercise prescription. A HIIT label alone was not considered sufficient for classification. Each study's prescribed or monitored intensity was extracted and appraised against youth guidance (approximately 85%–95% HRmax or 80%–100% peak work rate) (20). Studies were retained only when the primary report provided at least one of these indicators of high-intensity exercise.

Eligible comparators included usual school activity, usual PE, no additional intervention, a wait-list or inactive control, lower-intensity physical activity, moderate-intensity continuous training, or another active exercise condition. Eligible outcomes included physical fitness, body composition, cardiovascular or metabolic indicators, cognitive function, mental health, academic performance, attendance, classroom engagement, feasibility, fidelity, acceptability, adherence, and safety. These outcome domains were selected to capture both the direct physical and physiological effects of school-based HIIT and the cognitive, psychological, educational, and implementation-related outcomes that are relevant when considering its use in school settings. Randomised, cluster-randomised, quasi-experimental, and controlled intervention studies were eligible. Protocols, conference abstracts without sufficient outcome data, reviews, qualitative-only studies, cross-sectional studies, studies outside a school setting, and studies outside the school-aged population were excluded. The review was limited to English-language reports published from January 2010 to May 9, 2026 to focus on contemporary school-based HIIT formats; the language restriction reflected the review team's available translation resources.

### 2.3 Information sources and search strategy

Web of Science Core Collection, MEDLINE (Ovid), PubMed, PsycINFO, and ProQuest were searched, with the final searches completed on May 9, 2026. Reference lists of included studies and relevant reviews were also screened. Search concepts covered children, HIIT and related interval-training terminology, school settings, and the prespecified physical, psychological, cognitive, and academic outcome domains. Controlled vocabulary was combined with title, abstract, and subject-field terms as appropriate for each database. No restrictive study-design filter was applied because cluster-randomised and quasi-experimental school studies might otherwise have been missed. The complete database-specific search strategies, search dates, limits, and database-level record counts are provided in Supplementary File 1. The executed strategies included outcome-related terms; together with the English-language and date restrictions, this may have reduced search sensitivity. Reference-list screening identified no additional eligible study. Three potentially eligible reports could not be retrieved using the resources documented in the review archive. The corresponding authors of all three reports were contacted, but the full texts could not be obtained.

### 2.4 Study selection

All records were exported to EndNote. Duplicate records were removed using automated matching followed by manual checking. Two reviewers (LZY and LPL) independently screened titles and abstracts against the prespecified criteria. Reports judged potentially eligible by either reviewer proceeded to full-text assessment. The same reviewers independently assessed full texts, with disagreements resolved through discussion and consensus. Full-text exclusion reasons were recorded using prespecified categories. A citation-level list of all 88 full-text exclusions and their specific reasons is provided in Supplementary File 2.

### 2.5 Data collection and data items

Two reviewers (LZY and LPL) independently extracted data into a structured form and reconciled discrepancies through discussion. Extracted items included publication details; country and design; school type; participant number, age, and sex; intervention setting and delivery agent; HIIT modality; work and recovery intervals; intensity prescription and monitoring; session duration and frequency; programme duration; comparator; outcome definitions and assessment time points; adherence, fidelity, acceptability, enjoyment, adverse events, and implementation barriers; and results for each prespecified outcome domain. Intervention descriptions were interpreted using the Template for Intervention Description and Replication (TIDieR) principles to distinguish what was delivered, by whom, how often, at what intensity, and with what adaptations (6).

### 2.6 Risk of bias and certainty of evidence

Two reviewers (LZY and ZML) independently assessed risk of bias using the revised Cochrane risk-of-bias tool for randomised trials (RoB 2), with disagreements resolved by consensus (11). Judgements covered the randomisation process, deviations from intended interventions, missing outcome data, outcome measurement, selection of the reported result, and overall risk of bias. Because the interventions, comparators, and outcome constructs were too heterogeneous to define coherent pooled outcome bodies, formal GRADE certainty ratings were not undertaken. Instead, a concise GRADE-informed narrative assessment was used separately for physical fitness, cardiovascular outcomes, cognition, and implementation-related outcomes, considering risk of bias, heterogeneity, imprecision, and the amount of available evidence. This decision is considered when interpreting the strength of the conclusions. RoB 2 was used because all six included reports were treated as randomised or cluster-randomised trials; a non-randomised study would have required a design-appropriate tool such as ROBINS-I. No formal certainty-of-evidence rating was produced, so the evidence is described by its observed limitations rather than assigned an unsupported certainty category. Full domain-by-domain RoB 2 judgements for each included trial are presented in Supplementary File 3.

### 2.7 Synthesis method

Meta-analysis was not undertaken because studies differed substantially in participant characteristics, intervention modality and dose, comparator condition, outcome constructs, and reporting metrics. Studies were grouped by delivery model and outcome domain: physical fitness and movement performance; cardiovascular and physiological outcomes; cognition and school-relevant outcomes; and implementation, feasibility, and safety. No outcome domain was designated as primary; rather, the review aimed to map the outcomes assessed across the included studies within these prespecified domains. The synthesis reports the direction and consistency of findings without treating statistical significance as the sole indicator of importance, following the Synthesis Without Meta-analysis (SWiM) reporting guideline (12). For each result, the synthesis distinguished within-group pre–post change from between-group or group-by-time evidence wherever the primary report permitted this distinction.

## 3 Results

### 3.1 Study selection

The database searches identified 3,508 records: 731 from Web of Science, 352 from MEDLINE (Ovid), 336 from PubMed, 268 from PsycINFO, and 1,821 from ProQuest. After removal of 1,370 duplicates, 2,138 records were screened by title and abstract and 2,044 were excluded. Ninety-four full-text reports were assessed; 88 were excluded because of the study design (*n* = 29), intervention (*n* = 27), population (*n* = 18), language (*n* = 4), setting (*n* = 4), unavailable full text (*n* = 3), or comparator (*n* = 3). Six studies were included in the narrative synthesis (Figure 1). Reference-list screening yielded no additional eligible study.

**Figure 1:**
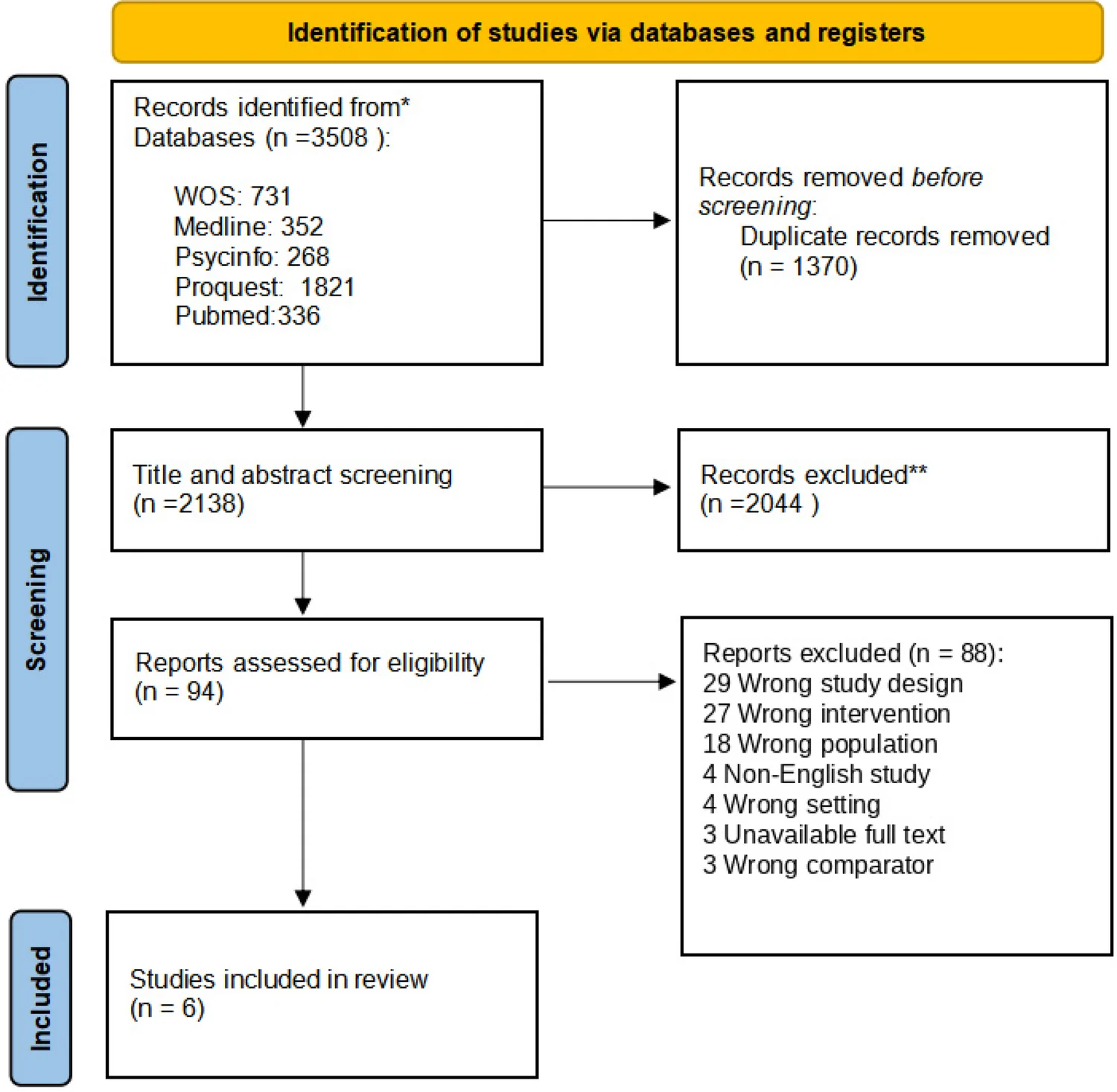
PRISMA 2020 flow diagram for study identification and selection.

### 3.2 Characteristics of included studies

The six studies were conducted in Germany, France, China, and the United States and represented 266 participants in the analysed samples. All included participants were primary-school-aged children, approximately 9–12 years old; no adolescent sample was represented. Interventions ranged from classroom-based body-weight micro-sessions to PE-based multi-activity protocols, running intervals, rope-skipping HIIT, and fitness- and skill-based circuits. Programme duration ranged from 4 to 12 weeks, session frequency from two to five sessions per week, and planned session time from 6 to 20 min. The interventions differed in both the physiological stimulus delivered and the outcomes selected (Table 1).

**Table 1:** Characteristics and principal findings of the included studies.

Study; country; design	Analysed sample; age; sex	HIIT intervention	Comparator	Dose	Principal findings	Overall RoB 2 judgement
Engel et al. (13); Germany; randomised feasibility study	35; 11–12 years; 24 boys and 11 girls	Classroom functional HIIT body-weight circuit	Usual classes	6 min; 4 d/wk; 4 wk; target RPE >17; pilot HR check in 10 children exceeded 85% HRmax	Fitness/movement: group-by-time effects favoured HIIT for lateral jumps (partial *η*^2^ = 0.382; *p* < 0.001), sit-ups (partial *η*^2^ = 0.485; *p* < 0.001), and 20-m sprint (partial *η*^2^ = 0.691; *p* < 0.001); no interaction for the 6-min run (partial *η*^2^ = 0.008; *p* = 0.615). Adherence: 86% of planned sessions were completed.	Some concerns
Ketelhut et al. (14); Germany; parallel-arm randomised trial	46; 10–12 years; sex NR	Multi-activity HIIT during the first 20 min of PE	Regular PE	20 min; 2 d/wk; 3 months; maximal/near-maximal effort; HR monitored weekly; numerical threshold NR	Fitness/cardiovascular: adjusted between-group changes favoured HIIT for aerobic fitness (7.73 *z*-score points; *p* = 0.007), peripheral systolic BP (−6.13 mmHg; *p* = 0.038), central systolic BP (−5.19 mmHg; *p* = 0.041), augmentation pressure (−2.02 mmHg; *p* = 0.013), and aPWV (−0.19 m/s; *p* = 0.031).	High risk
Ketelhut et al. (17); Germany; randomised controlled trial	40 analysed of 46 randomised; mean age 11 ± 1 years; about 44% girls and 56% boys	Child-specific HIIT during the first 20 min of PE	Regular PE	20 min; 2 d/wk; 12 wk; maximal/near-maximal effort prescribed; bout-level physiological threshold NR	Cardiovascular: group-by-time interactions favoured HIIT for aPWV (*η*^2^ = 0.099; *p* = 0.050), RMSSD (*η*^2^ = 0.161; *p* = 0.010), low-frequency power (*η*^2^ = 0.165; *p* = 0.009), high-frequency power (*η*^2^ = 0.272; *p* < 0.001), and LF/HF ratio (*η*^2^ = 0.354; *p* < 0.001); blood-pressure effects were not consistently different between groups.	High risk
Liu et al. (18); China; three-group randomised trial	60; 9–10 years; 33 boys and 27 girls	Rope-skipping HIIT: five 1-min bouts with 1-min recovery	MICT rope skipping and usual activity	Five 1-min bouts with 1-min recovery; ≥85% HRmax; 5 d/wk; 8 wk	Cognition: both HIIT and MICT showed pre–post reductions in incongruent Stroop reaction time and left-DLPFC oxyhaemoglobin activation; the report did not establish a HIIT-specific advantage over MICT.	High risk
Mucci et al. (15); France; two-arm randomised study	18; mean age 10.1 ± 0.7 years; 8 girls and 10 boys	Running-based interval training added to PE	No additional training	Four sets of ten 10–20-s shuttle runs at 100%–130% MAS with equal recovery; 2 d/wk; 8 wk	Fitness: within-group changes in HIIT were VT1 + 4.35 ± 4.36 mL O_2_·min^−1^·kg^−1^ (21%), VT2 + 7.17 ± 5.17 mL O_2_·min−1·kg−1 (24%), and VO2peak +5.51 ± 4.17 mL O_2_·min−1·kg−1 (14%) (all *p* < 0.01); the control group showed no significant pre–post change. A directly estimated between-group effect was NR.	Some concerns
Ricci et al. (16); United States; randomised pilot evaluation	67; mean age 10.5 ± 0.9 years; 42 girls and 25 boys	Fitness- and skill-based HIIT circuit at the start of PE	Regular PE	16–19 min; 8 sessions over 6 wk; accelerometry documented vigorous activity; bout-level physiological threshold NR	Implementation/fitness: satisfaction was 3.6 ± 1.5/5 and teacher feasibility was 31/35. Vigorous activity was greater with HIIT than regular PE (4.5 ± 2.6 vs. 2.3 ± 1.3 min/h; partial *η*^2^ = 0.27). VO2peak changed from 53.6 ± 6.1 to 56.9 ± 7.3 mL/kg/min in HIIT and from 53.9 ± 7.0 to 52.4 ± 10.4 mL/kg/min in controls (partial *η*^2^ = 0.26).	Some concerns

aPWV, aortic pulse-wave velocity; BP, blood pressure; d/wk, days per week; DLPFC, dorsolateral prefrontal cortex; HIIT, high-intensity interval training; HR, heart rate; HRmax, maximal heart rate; MAS, maximal aerobic speed; MICT, moderate-intensity continuous training; min, minutes; NR, not reported; PE, physical education; RoB 2, revised Cochrane risk-of-bias tool for randomised trials; RPE, rating of perceived exertion; s, seconds; VO2peak, peak oxygen uptake; VT, ventilatory threshold; wk, weeks; *η*^2^, eta squared. In the Principal findings column, statistics are presented, where available, in the order of effect or change, variability or effect size, and *p* value; NR indicates not reported in the primary report.

### 3.3 Physical fitness and movement performance

Evidence for physical-fitness outcomes was mixed but more developed than evidence for school-relevant outcomes. In a 4-week classroom feasibility trial, Functional HIIT did not improve 6-minute run performance but produced greater improvements in lateral jumps, sit-ups, and 20-m sprint performance than the control condition (13). The multi-activity PE trial reported adjusted between-group changes favouring HIIT for aerobic fitness and selected vascular outcomes (14), whereas the running-based study reported within-group improvements in ventilatory thresholds and VO2peak without a directly estimated between-group effect (15). The corresponding overall RoB 2 judgements were some concerns for Engel et al. (13), high risk for Ketelhut et al. (14), and some concerns for Mucci et al. (15).

The fitness- and skill-based PE programme evaluated by Ricci et al. (16) was designed to preserve students' PE experience while increasing vigorous activity. It produced favourable feasibility and satisfaction findings and a pattern of higher vigorous activity during PE. Nevertheless, the evidence is too small and heterogeneous to identify a single optimal school-based HIIT format or to support a universal effect on cardiorespiratory fitness. Ricci et al. (16) was judged as having some concerns overall.

### 3.4 Cardiovascular and physiological outcomes

Two German trials reported cardiovascular or haemodynamic outcomes. Ketelhut et al. (14) found effects favouring multi-activity HIIT for aerobic fitness, peripheral and central systolic blood pressure, augmentation pressure, and aortic pulse-wave velocity. Ketelhut et al. (17) reported improvements in aortic pulse-wave velocity and several heart-rate-variability indices, whereas blood-pressure findings were not consistently different between groups. These findings arose from only two small trials in generally healthy children, and the measurement context is sensitive to maturation, sleep, diet, and physical activity outside school. The cardiovascular findings should therefore be interpreted as preliminary and protocol-dependent. Both cardiovascular trials were judged at high overall risk of bias (14, 17).

### 3.5 Cognitive and school-relevant outcomes

Only one included study directly examined cognition. Liu et al. (18) compared 8 weeks of rope-skipping HIIT, moderate-intensity continuous training, and usual activity in children aged 9–10 years. Both exercise groups showed faster reaction times in the incongruent condition of a Stroop task and lower left dorsolateral prefrontal cortex oxyhaemoglobin activation, interpreted as improved neural efficiency. Because both exercise conditions improved, the study does not demonstrate a HIIT-specific cognitive advantage. No included study directly assessed depression, anxiety, wellbeing, mathematics or literacy achievement, attendance, or classroom engagement. The evidence therefore does not support claims that school-based HIIT improves mental health or academic performance. The cognition trial was judged at high overall risk of bias (18).

### 3.6 Implementation, feasibility, and safety

Implementation outcomes were reported inconsistently. Engel et al. (13) showed that brief classroom micro-sessions could be delivered four days per week, with 86% session completion and mean perceived exertion consistent with vigorous exercise. Ricci et al. (16) incorporated fitness and skill elements into PE and reported feasibility and satisfaction data. Safety reporting also varied across studies. Ketelhut et al. (14) reported no notifiable accidents or adverse events in either group, Ricci et al. (16) reported no noticeable adverse events or injuries observed or reported during the intervention, and Ketelhut et al. (17) reported no adverse events and explicitly defined adverse events to include injuries, nausea, vomiting, dizziness, or other physical or discomfort-related events preventing completion of an exercise session. In contrast, Engel et al. (13), Mucci et al. (15), and Liu et al. (18) did not report intervention-related adverse-event outcomes or describe adverse-event monitoring procedures; therefore, absence of reported events in these studies should not be interpreted as evidence that no adverse events occurred. Most reports, however, did not provide a complete account of teacher training, fidelity, adaptation for different abilities, adverse-event monitoring, or the practical burden placed on schools. This limitation mirrors the wider school-based HIIT literature, in which process-evaluation reporting is frequently incomplete (8). Given the incomplete reporting of standardised adverse-event monitoring procedures and process-evaluation indicators, the absence of reported adverse events should be interpreted cautiously, and the feasibility and consistency of implementation in routine school settings remain uncertain.

### 3.7 Risk of bias

All six studies were judged to have at least some risk-of-bias concerns. Engel et al. (13), Mucci et al. (15), and Ricci et al. (16) were rated as having some concerns overall, whereas Ketelhut et al. (14), Ketelhut et al. (17), and Liu et al. (18) were rated at high overall risk. The most consistent issue was selection of the reported result, for which all six studies raised some concerns. Five studies raised concerns in the randomisation domain, two were at high risk because of deviations from intended interventions, and five raised concerns about missing outcome data. Outcome measurement was comparatively stronger, with four studies rated at low risk and two raising some concerns (Figure 2). Study-specific overall judgements are also reported in Table 1. Because no study was rated at low overall risk of bias, none of the favourable outcome findings was interpreted as low-risk evidence.

**Figure 2:**
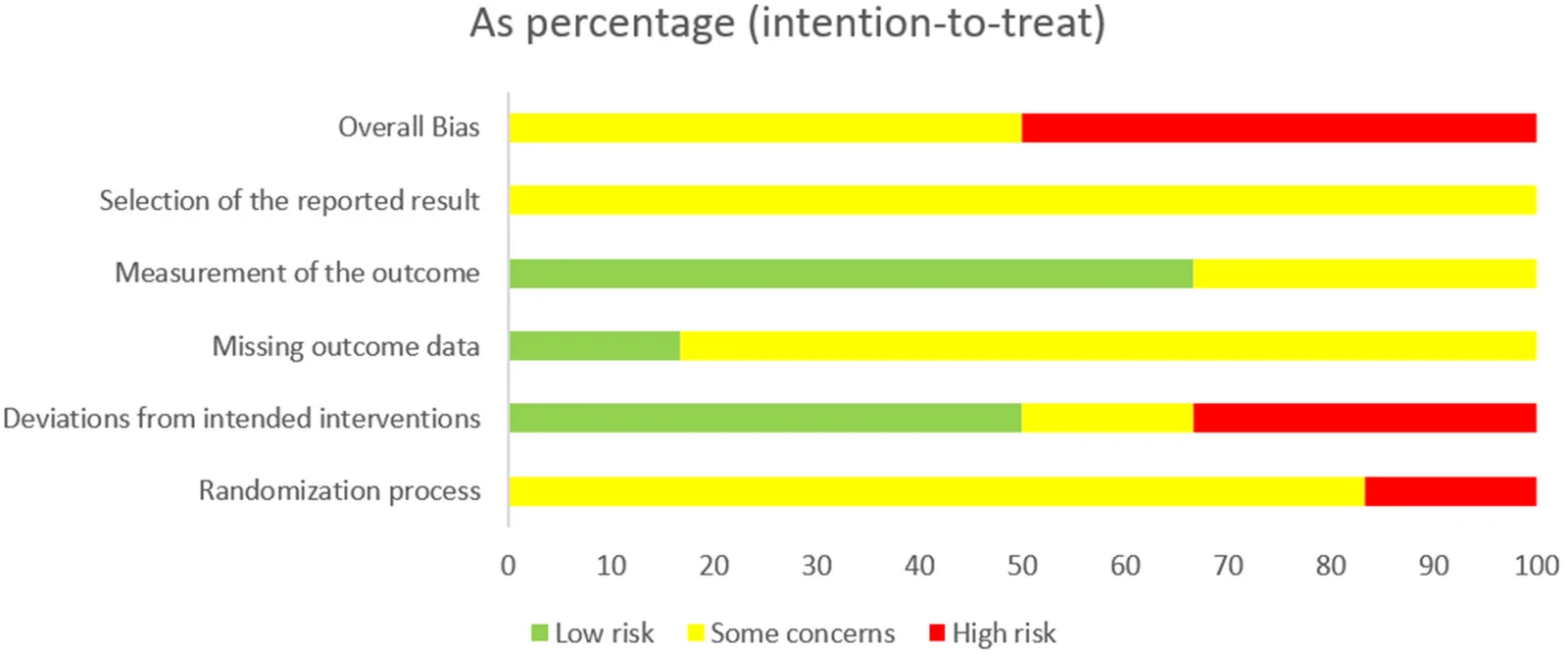
Aggregate RoB 2 judgements across the six included studies, shown as percentages by domain.

## 4 Discussion

### 4.1 Principal findings

This focused review indicates that school-based HIIT can be delivered in several practical forms and that some studies reported favourable findings for selected physical-fitness, movement, and cardiovascular outcomes. The pattern was not uniform. Short classroom circuits appeared more likely to improve movement-specific outcomes than aerobic endurance, whereas PE-based or running-based protocols that accumulated more aerobic work were better positioned to influence cardiorespiratory outcomes. The current evidence provides only preliminary support for cardiovascular adaptation and does not establish benefits for mental health, academic performance, attendance, or classroom engagement. Similarly, the single cognition study should not be interpreted as evidence of a HIIT-specific cognitive benefit, because comparable improvements were also observed with moderate-intensity continuous training. Moreover, no included studies assessed mental health, academic performance, attendance, or classroom engagement; therefore, no conclusions can currently be drawn regarding the effects of school-based HIIT on these outcomes. These findings should be interpreted cautiously because all six included studies had risk-of-bias concerns and the available evidence base was small and heterogeneous. Although a formal GRADE assessment was not undertaken, the narrative certainty assessment was considered separately across outcome domains. Confidence in the physical-fitness findings is limited because results were inconsistent across small, heterogeneous studies with risk-of-bias concerns. Confidence in the cardiovascular findings is particularly limited because the evidence came from only two small trials, both at high overall risk of bias. Confidence in the cognitive findings is very limited because only one high-risk study assessed cognition and did not demonstrate a HIIT-specific advantage. Confidence in the implementation-related findings is also limited because feasibility, fidelity, acceptability, and adverse-event reporting were incomplete and inconsistent. Overall, the small evidence base limits confidence in the overall findings, and the results should therefore be interpreted cautiously. The apparent alignment between movement-specific training and movement-test findings may reflect task specificity, but the included studies did not test this mechanism directly.

Generalizability is limited by the age range represented. All participants were approximately 9–12 years old, and no adolescent sample was represented. The findings should therefore not be extrapolated to adolescents or younger primary-school children.

The distinction between measured and aspirational outcomes is central. HIIT is often promoted as a strategy that might improve health, wellbeing, cognition, and learning while saving school time. The included evidence does not justify combining these claims. A school may reasonably consider a brief HIIT programme as one option for increasing vigorous activity and targeting selected fitness outcomes, but HIIT should not be presented as a replacement for comprehensive PE or as a proven academic or mental-health intervention. Comprehensive PE also serves educational, motor-skill, social, and enjoyment-related functions that a brief interval session cannot replace.

### 4.2 Positioning within the wider evidence base

The findings should be interpreted alongside recent comprehensive syntheses. Duncombe et al. (7) identified 42 school-based HIIT interventions and found that physical and cardiometabolic outcomes could improve compared with control conditions, although many studies had a high risk of bias and comparisons with other exercise modes were mixed. Liu et al. (8) subsequently identified 45 interventions and showed that implementation reporting was commonly incomplete. The contribution of the present focused review is therefore not to replace these broader syntheses, but to emphasise the alignment between intervention claims, directly measured outcomes, and implementation requirements in schools.

### 4.3 Implications for practice

Given the limited and heterogeneous evidence base, school-based HIIT may be considered as a brief, adaptable supplement to PE or classroom activity opportunities when schools aim to increase vigorous activity or target selected fitness-related outcomes. Programme design should specify the intended intensity, work and recovery intervals, progression, adaptations for different abilities, teacher preparation, safety procedures, and monitoring plan. Enjoyment, choice, social climate, and teacher workload should be treated as implementation outcomes rather than optional extras. Low-equipment formats may facilitate delivery, but feasibility must be demonstrated within the local timetable and school culture. Although individual HIIT sessions were relatively brief, short session duration should not be interpreted as evidence of school-level time efficiency, because implementation burden, staff time, and other resource requirements were rarely or incompletely reported.

### 4.4 Implications for future research

Future school-based trials should use adequately powered cluster-randomised designs when delivery occurs by class or school, account for clustering in the analysis, and report according to the CONSORT extension for cluster trials (21). Intervention reporting should enable replication through TIDieR-consistent descriptions of exercise mode, intensity target, work-to-rest structure, dose, facilitator, resources, adaptations, fidelity, attendance, adverse events, and student experience. Future trials should also report session- and bout-level intensity monitoring to verify that the prescribed HIIT intensity is achieved, rather than relying on nominal HIIT labelling alone. Researchers should prespecify a small set of outcomes aligned with the theory of change. When HIIT is proposed as a mental-health or academic intervention, validated mental-health and academic measures, attendance, engagement, and process outcomes should be measured alongside fitness.

### 4.5 Strengths and limitations

Strengths of this review include its explicit focus on school relevance, structured PICOS criteria, independent two-reviewer procedures, detailed consideration of intervention delivery, and transparent narrative synthesis. It also separates directly measured outcomes from broader claims about wellbeing and learning.

Several limitations require consideration. The search was restricted to English-language reports published from January 2010 to May 9, 2026 and included outcome-related terms; each of these restrictions may have reduced search sensitivity and may have excluded otherwise eligible studies captured by broader school-based HIIT reviews. Only six studies were included, samples were small, interventions were short, and outcome measures were heterogeneous. Meta-analysis was not appropriate, and formal GRADE ratings were not undertaken. Risk-of-bias concerns affected all six studies, with three studies judged at high overall risk and three as having some concerns; no study was rated at low overall risk. Study-specific overall judgements are reported in Table 1, while Figure 2 retains the aggregate domain-level profile. In addition, several included studies did not provide session- or bout-level verification that the prescribed high-intensity stimulus was consistently achieved; therefore, nominal classification as HIIT may not always represent confirmed exposure at the recommended intensity. Finally, incomplete reporting of allocation, fidelity, adverse events, and implementation processes restricted assessment of how readily the interventions could be transferred to routine school practice. Three potentially eligible reports were unavailable despite attempts to contact the corresponding authors of all three reports, creating possible availability bias. Given the small evidence base, these unretrieved reports may have affected the completeness of the available evidence and therefore further reduced confidence in the overall conclusions. The included evidence was limited to children; no adolescents were represented, so applicability to adolescents is unknown.

## 5 Conclusion

School-based HIIT can be delivered in brief formats within classroom and PE settings, but the current focused evidence base is small, heterogeneous, and affected by risk-of-bias concerns. No included study achieved a low overall risk-of-bias rating; all observed favourable outcomes are supported by evidence judged as either high risk or some concerns. Preliminary findings suggest possible benefits for selected physical-fitness, movement, and cardiovascular outcomes, although these effects were not consistent across interventions and should not be interpreted as evidence of established effectiveness. Evidence for a HIIT-specific cognitive benefit remains limited, and the included studies provide no direct evidence regarding mental health, academic performance, attendance, or classroom engagement. Furthermore, the short duration of individual HIIT sessions should not be equated with demonstrated time efficiency because implementation burden and resource requirements were incompletely reported. Future school-based trials should use adequately powered designs and provide stronger reporting of intervention intensity, safety, fidelity, adaptation, feasibility, and school-relevant outcomes before firm conclusions regarding effectiveness, applicability, or efficiency can be drawn. These conclusions apply only to the primary-school-aged children represented in the included studies and should not be generalised to adolescents.
