# Supplementary material for: School-based high-intensity interval training for children: a focused systematic review of implementation and school-relevant outcomes
Source: Front Pediatr. 2026 Sep 28;14:1952928. doi: 10.3389/fped.2026.1952928 (PMC13655682; doi:10.3389/fped.2026.1952928)
Supplement: Supplementary file 1 [file Supplementaryfile1.docx]

**Supplementary File 1. Database-specific Search Strategies and Search Results**

Final searches were completed on May 9, 2026. The review was limited to English-language reports published from January 2010 to May 9, 2026. No restrictive study-design filter was applied. Search strings are reproduced as used in each database interface.

# Search results summary

| **Database/platform** | **Records retrieved** |
| --- | --- |
| Web of Science Core Collection | 731 |
| MEDLINE (Ovid) | 352 |
| PubMed | 336 |
| PsycINFO | 268 |
| ProQuest | 1,821 |
| **Total** | **3,508** |

# Database-specific strategies

## PubMed

Fields: title/abstract. Interface limits: English; publication date January 2010–May 9, 2026. Records retrieved: 336.

( "high-intensity interval training"[Title/Abstract] OR "high intensity interval training"[Title/Abstract] OR HIIT[Title/Abstract] OR "Sprint Interval Training"[Title/Abstract]) AND ("school-based"[Title/Abstract] OR "school based"[Title/Abstract] OR school*[Title/Abstract] OR student*[Title/Abstract] OR "adolescent"[Title/Abstract] OR adolescents[Title/Abstract] OR child*[Title/Abstract] OR youth[Title/Abstract]) AND ("mental health"[Title/Abstract] OR "psychological well-being"[Title/Abstract] OR "psychological health"[Title/Abstract] OR "depression"[Title/Abstract] OR "anxiety"[Title/Abstract] OR "stress"[Title/Abstract] OR "physical fitness"[Title/Abstract] OR "physical activity"[Title/Abstract] OR "cardiorespiratory fitness"[Title/Abstract] OR "endurance"[Title/Abstract] OR "aerobic capacity"[Title/Abstract] OR "muscular strength"[Title/Abstract] OR "academic performance"[Title/Abstract] OR "academic achievement"[Title/Abstract] OR "learning outcome*"[Title/Abstract])

## Web of Science Core Collection

Field: Topic (TS). Interface limits: English; publication date January 2010–May 9, 2026. Records retrieved: 731.

TS=(("high-intensity interval training" OR "HIIT" OR "high intensity interval exercise" OR "high-intensity intermittent exercise") AND( "school-based" OR "school based" OR "school" OR "student" OR "adolescent" OR "youth" OR "children" OR "pupil" ) AND ("mental health" OR "psychological health" OR "well-being" OR "wellbeing" OR "depression" OR "anxiety" OR "stress" OR "self-esteem" OR "self-efficacy" OR "mood" OR "physical fitness" OR "physical activity" OR "aerobic fitness" OR "cardiorespiratory fitness" OR "muscular fitness" OR "muscle strength" OR "body composition" OR "endurance" OR "academic performance" OR "academic achievement" OR "cognitive function" OR "executive function" OR "learning" OR "grades" OR "school performance" ))

## MEDLINE (Ovid)

Platform: Ovid. Interface limits: English; publication date January 2010–May 9, 2026. Records retrieved: 352.

("Child") AND ("Schools") AND ("Exercise" OR "Sports") AND ("Mental Health" OR "Physical Fitness" OR "Academic Performance").

## PsycINFO

Fields: title, abstract, subject (ti,ab,su). Interface limits: English; publication date January 2010–May 9, 2026. Records retrieved: 268.

ti,ab,su(school* OR student* OR pupil* OR adolescent* OR child* OR youth* OR teen* OR "physical education" OR PE) AND ti,ab,su("high intensity interval training" OR "high-intensity interval" OR HIIT OR HIIE OR "high intensity interval exercise" OR "sprint interval training" OR SIT OR "high intensity circuit training" OR HICT OR "interval training") AND ti,ab,su("mental health" OR "mental well-being" OR "mental wellbeing" OR psychological OR depression OR anxiety OR stress OR mood OR "executive function" OR cognition OR "cognitive function" OR "physical fitness" OR "cardiorespiratory fitness" OR "cardiovascular fitness" OR "physical health" OR "body composition" OR BMI OR obesity OR "aerobic capacity" OR VO2max OR "academic performance" OR "academic achievement" OR "school performance" OR grades OR GPA OR "academic success" OR "educational attainment")

## ProQuest

Field: anywhere except full text (noft). Interface limits: English; publication date January 2010–May 9, 2026. Records retrieved: 1,821.

noft(( "school " OR " student " OR "pupil " OR " adolescent " OR "child "OR "youth "OR "teen ")) AND noft(("high intensity interval training" OR "high-intensity interval" OR HIIT OR HIIE OR "high intensity interval exercise" OR "sprint interval training" OR SIT OR "high intensity circuit training" OR HICT OR "interval training")) AND noft(("mental health" OR "mental well-being" OR "mental wellbeing" OR psychological OR depression OR anxiety OR stress OR mood OR "executive function" OR cognition OR "cognitive function" OR "physical fitness" OR "cardiorespiratory fitness" OR "cardiovascular fitness" OR "physical health" OR "body composition" OR BMI OR obesity OR "aerobic capacity" OR VO2max OR "academic performance" OR "academic achievement" OR "school performance" OR grades OR GPA OR "academic success" OR "educational attainment"))
