# Supplementary material for: School-based high-intensity interval training for children: a focused systematic review of implementation and school-relevant outcomes
Source: Front Pediatr. 2026 Sep 28;14:1952928. doi: 10.3389/fped.2026.1952928 (PMC13655682; doi:10.3389/fped.2026.1952928)
Supplement: Supplementary file 2 [file Supplementaryfile2.docx]

**Supplementary File 2**

**Citation-level Full-text Exclusions and Reasons for Exclusion**

**Scope note.** The prespecified participant age range was 5–12 years. The table below lists all 88 reports excluded after full-text assessment. Exclusion categories have been harmonised to the PRISMA categories used in Figure 1, while the specific reason reflects the screening record.

**PRISMA full-text exclusion summary**

| **Category** | **n** |
| --- | --- |
| Study design | 29 |
| Intervention | 27 |
| Population | 18 |
| Language | 4 |
| Setting | 4 |
| Unavailable full text | 3 |
| Comparator | 3 |
| **Total** | **88** |

**Citation-level exclusion list**

| **No.** | **Citation** | **DOI / identifier** | **PRISMA category** | **Specific reason for exclusion** |
| --- | --- | --- | --- | --- |
| 1 | Agurto 2018 — Efficacy of a High-Intensity Interval Training program in corporal variables modification on preadolescent schoolchildren of a school in the city of Temuco, Chile. Revista Espanola De Nutricion Humana Y Dietetica (2018). | 10.14306/renhyd.22.2.448 | Language | Report was not published in English. |
| 2 | Alpert 1990 — Aerobics enhances cardiovascular fitness and agility in preschoolers. Health Psychology (1990). | https://dx.doi.org/10.1037/0278-6133.9.1.48 | Population | Participant population was younger than the prespecified lower age limit of 5 years. |
| 3 | AnaCarolinaCortede 2012 — Similar Health Benefits of Endurance and High-Intensity Interval Training in Obese Children. PLoS One (2012). | https://doi.org/10.1371/journal.pone.0042747 | Setting | Intervention was not delivered in an eligible school setting. |
| 4 | Andrade-Lara 2023 — Can 16 Minutes of HIIT Improve Attentional Resources in Young Students?. Journal of Functional Morphology and Kinesiology (2023). | 10.3390/jfmk8030116 | Intervention | Intervention did not meet the prespecified HIIT eligibility criterion. |
| 5 | Aparecido 2022 — Six HIT Sessions Improve Cardiorespiratory Fitness and Metabolic Flexibility in Insulin Resistant and Insulin Sensitive Adolescents with Obesity. International Journal of Environmental Research and Public Health (2022). | 10.3390/ijerph191710568 | Study design | Study did not use an eligible controlled intervention design. |
| 6 | Brustio 2023 — Commentary: Comment and reflection about Mental health outcomes of the Daily Mile in elementary school children: a single-arm pilot study. The implementation of the Daily Mile in the school context. Child & Adolescent Mental Health (2023). | https://dx.doi.org/10.1111/camh.12606 | Study design | Ineligible study design. |
| 7 | Bueno 2021 — Association Between Device-Measured Moderate-to-Vigorous Physical Activity and Academic Performance in Adolescents. Health Education & Behavior (2021). | https://dx.doi.org/10.1177/1090198120954390 | Study design | Cross-sectional study; no eligible intervention comparison. |
| 8 | Burford 2022 — Children's Enjoyment, Perceived Competency, and Vigorous Physical Activity during High-Intensity Interval Training in Physical Education. Research Quarterly for Exercise and Sport (2022). | 10.1080/02701367.2021.1925207 | Study design | Study did not use an eligible controlled intervention design. |
| 9 | Burrows 2014 — Scheduled physical activity is associated with better academic performance in Chilean school-age children. Journal of Physical Activity & Health (2014). | https://dx.doi.org/10.1123/jpah.2013-0125 | Study design | Study did not use an eligible controlled intervention design. |
| 10 | Cao 2022 — Effects of school-based high-intensity interval training on body composition, cardiorespiratory fitness and cardiometabolic markers in adolescent boys with obesity: a randomized controlled trial. BMC Pediatrics (2022). | 10.1186/s12887-021-03079-z | Population | Participant population was older than the prespecified upper age limit of 12 years. |
| 11 | Cao 2022 — Integrating High-Intensity Interval Training into a School Setting Improve Body Composition, Cardiorespiratory Fitness and Physical Activity in Children with Obesity: A Randomized Controlled Trial. J Clin Med (2022). | 10.3390/jcm11185436 | Population | Participant population was older than the prespecified upper age limit of 12 years. |
| 12 | Chandler 2020 — A Comprehensive Exploration into Utilizing High-Intensity Interval Training (HIIT) in Physical Education Classes. Journal of Physical Education Recreation and Dance (2020). | 10.1080/07303084.2019.1679296 | Study design | Ineligible study design. |
| 13 | Chuensiri 2018 — Effects of High-Intensity Intermittent Training on Vascular Function in Obese Preadolescent Boys. Child Obes (2018). | 10.1089/chi.2017.0024 | Population | Clinical/unhealthy participant population outside the target school population. |
| 14 | Chuensiri 2015 — The Acute Effects of Supramaximal High-Intensity Intermittent Exercise on Vascular Function in Lean vs. Obese Prepubescent Boys. Pediatric Exercise Science (2015). | 10.1123/pes.2015-0100 | Study design | Ineligible study design. |
| 15 | Cockcroft 2018 — A single bout of high-intensity interval exercise and work-matched moderate-intensity exercise has minimal effect on glucose tolerance and insulin sensitivity in 7-to 10-year-old boys. Journal of Sports Sciences (2018). | 10.1080/02640414.2017.1287934 | Intervention | Intervention did not meet the prespecified HIIT eligibility criterion. |
| 16 | deFreitas 2024 — Utility of a high-intensity interval training app as a remote exercise support strategy in children with obesity: An exploratory study of adherence, effect, and perceptions of its use. Digital Health (2024). | 10.1177/20552076241291386 | Study design | Ineligible study design. |
| 17 | deMenezes 2026 — Individual response to continuous and interval training on cognitive and brain-derived neurotrophic factor in adolescents with overweight/obesity. Pediatric Research (2026). | 10.1038/s41390-025-04737-z | Intervention | Intervention did not meet the prespecified HIIT eligibility criterion. |
| 18 | Delgado-Floody 2018 — Effects of 28 weeks of high-intensity interval training during physical education classes on cardiometabolic risk factors in Chilean schoolchildren: a pilot trial. European Journal of Pediatrics (2018). | 10.1007/s00431-018-3149-3 | Study design | Study did not use an eligible controlled intervention design. |
| 19 | Dias 2018 — Effect of High-Intensity Interval Training on Fitness, Fat Mass and Cardiometabolic Biomarkers in Children with Obesity: A Randomised Controlled Trial. Sports Medicine (2018). | 10.1007/s40279-017-0777-0 | Intervention | Intervention did not meet the prespecified HIIT eligibility criterion. |
| 20 | Drollette 2024 — Take a Break for Memory Sake! Effects of Short Physical Activity Breaks on Inhibitory Control, Episodic Memory, and Event-Related Potentials in Children. Brain Sciences (2024). | 10.3390/brainsci14070626 | Setting | Intervention was not delivered in an eligible school setting. |
| 21 | Duncombe 2024 — Was it a HIIT? A process evaluation of a school-based high-intensity interval training intervention. International Journal of Behavioral Nutrition and Physical Activity (2024). | 10.1186/s12966-024-01599-2 | Study design | Ineligible study design. |
| 22 | Duncombe 2023 — Making a HIIT: co-design of high-intensity interval training workouts with students & teachers within the curriculum. BMC Public Health (2023). | 10.1186/s12889-023-16613-8 | Population | Adult participant population. |
| 23 | Eather 2026 — Exploring the Impact of Choice and Variety on Adolescents' Motivation to Participate in High-Intensity Interval Training During Physical Education: A Randomized Controlled Trial. Journal of Teaching in Physical Education (2026). | 10.1123/jtpe.2024-0173 | Population | Participant population was older than the prespecified upper age limit of 12 years. |
| 24 | Eggertsen 2025 — Feasibility and efficacy of adding high-intensity interval training to a multidisciplinary lifestyle intervention in children with obesity-a randomized controlled trial. International Journal of Obesity (2025). | 10.1038/s41366-024-01645-w | Intervention | Intervention did not meet the prespecified HIIT eligibility criterion. |
| 25 | Eggertsen 2025 — Adding High-Intensity Interval Training to a Multidisciplinary Lifestyle Intervention for Childhood Obesity: Secondary Analysis of a Randomized Controlled Trial to Examine the Effects on Physical Activity Behavior. Scandinavian Journal of Medicine & Science in Sports (2025). | 10.1111/sms.70185 | Intervention | Intervention did not meet the prespecified HIIT eligibility criterion. |
| 26 | Engel 2018 — High-intensity interval training in physical education. German Journal of Exercise and Sport Research (2018). | 10.1007/s12662-018-0492-5 | Language | Report was not published in English. |
| 27 | Espinoza-Silva 2019 — Response of obese schoolchildren to high-intensity interval training applied in the school context. Endocrinol Diabetes Nutr (Engl Ed) (2019). | 10.1016/j.endinu.2019.05.005 | Language | Report was not published in English. |
| 28 | Faigenbaum 2009 — "Plyo Play": A Novel Program of Short Bouts of Moderate and High Intensity Exercise Improves Physical Fitness in Elementary School Children. Physical Educator (2009). | 232994275 | Unavailable full text | Full text could not be retrieved from the resources documented in the review archive. |
| 29 | Feng 2022 — Association between vigorous physical activity and life satisfaction in adolescents. Frontiers in Public Health (2022). | https://dx.doi.org/10.3389/fpubh.2022.944620 | Study design | Cross-sectional study; no eligible intervention comparison. |
| 30 | Feng 2024 — RESEARCH ON EFFECTS OF HIGH INTENSITY INTERVAL TRAINING ON CARDIOPULMONARY FUNCTION IN ADOLESCENTS. Revista Internacional De Medicina Y Ciencias De La Actividad Fisica Y Del Deporte (2024). | 10.15366/rimcafd2024.95.003 | Study design | Ineligible study design. |
| 31 | Feng 2023 — Time course efficiency of MICE and HIIE on inhibitory control and HRV in adolescents with obesity and different cardiorespiratory fitness. Frontiers in Psychology (2023). | 10.3389/fpsyg.2023.1242190 | Study design | Ineligible study design. |
| 32 | Fernández 2017 — EFFECT OF AN INTERMITTENT HIGH INTENSITY TRAINING PROGRAM IN OVERWEIGHT AND OBESE CHILDREN IN ARICA, CHILE. Interciencia (2017). | WOS:000397171600007 | Unavailable full text | Full text could not be retrieved from the resources documented in the review archive. |
| 33 | Gelabert 2023 — The effects of a moderate-to-high intensity interval exercise training programme on selective and sustained attention in schoolchildren aged 11 and 12. Journal of Human Sport and Exercise (2023). | 10.14198/jhse.2023.181.17 | Study design | Uncontrolled single-group pre–post quasi-experimental design. |
| 34 | Gonçalves 2024 — Strategies in physical education and their impact on student intensity, perceptions, and engagement. Journal of Physical Education and Sport (2024). | https://doi.org/10.7752/jpes.2024.12319 | Unavailable full text | Full text could not be retrieved from the resources documented in the review archive. |
| 35 | Grasten 2022 — Developmental associations of accelerometer measured moderate-to-vigorous physical activity and sedentary time with cardiorespiratory fitness in schoolchildren. Journal of Science and Medicine in Sport (2022). | https://dx.doi.org/10.1016/j.jsams.2022.08.015 | Intervention | Intervention did not meet the prespecified HIIT eligibility criterion. |
| 36 | Harris 2021 — Feasibility and Provisional Efficacy of Embedding High-Intensity Interval Training Into Physical Education Lessons: A Pilot Cluster-Randomized Controlled Trial. Pediatric Exercise Science (2021). | 10.1123/pes.2020-0255 | Population | Participant population was older than the prespecified upper age limit of 12 years. |
| 37 | Hatch 2021 — Effect of Differing Durations of High-Intensity Intermittent Activity on Cognitive Function in Adolescents. International Journal of Environmental Research and Public Health (2021). | 10.3390/ijerph182111594 | Intervention | Intervention did not meet the prespecified HIIT eligibility criterion. |
| 38 | Have 2018 — Classroom-based physical activity improves children's math achievement - A randomized controlled trial. PLoS ONE [Electronic Resource] (2018). | https://dx.doi.org/10.1371/journal.pone.0208787 | Intervention | Intervention did not meet the prespecified HIIT eligibility criterion. |
| 39 | Huberty 2011 — Ready for recess: a pilot study to increase physical activity in elementary school children. Journal of School Health (2011). | https://dx.doi.org/10.1111/j.1746-1561.2011.00591.x | Study design | Study did not use an eligible controlled intervention design. |
| 40 | Ishihara 2020 — Relationship of participation in specific sports to academic performance in adolescents: A 2-year longitudinal study. Scandinavian Journal of Medicine & Science in Sports (2020). | https://dx.doi.org/10.1111/sms.13703 | Study design | Study did not use an eligible controlled intervention design. |
| 41 | Jarnig 2023 — Effects of a daily physical activity intervention on the health-related fitness status of primary school children: A cluster randomized controlled trial. Journal of Sports Sciences (2023). | https://dx.doi.org/10.1080/02640414.2023.2259210 | Intervention | Intervention did not meet the prespecified HIIT eligibility criterion. |
| 42 | JoséMiguelEspinoza 2023 — Effects of a High Intensity Interval Training (HIIT) Program on Anthropomorphic and Cardiometabolic Variables in School Children with Overweight and Obesity. Children (2023). | https://doi.org/10.3390/children10020317 | Study design | Study did not use an eligible controlled intervention design. |
| 43 | Jurak 2013 — Long-term effects of 4-year longitudinal school-based physical activity intervention on the physical fitness of children and youth during 7-year followup assessment. Central European Journal of Public Health (2013). | https://dx.doi.org/10.21101/cejph.a3823 | Intervention | Intervention did not meet the prespecified HIIT eligibility criterion. |
| 44 | Juric 2023 — Does incorporating high intensity interval training in physical education classes improve fitness outcomes of students? A cluster randomized controlled trial. Preventive Medicine Reports (2023). | 10.1016/j.pmedr.2023.102127 | Population | Participant population was older than the prespecified upper age limit of 12 years. |
| 45 | Kao 2023 — Acute effects of intense interval versus aerobic exercise on children's behavioral and neuroelectric measures of inhibitory control. Journal of Science and Medicine in Sport (2023). | 10.1016/j.jsams.2023.05.003 | Intervention | Intervention did not meet the prespecified HIIT eligibility criterion. |
| 46 | Kargarfard 2016 — Effects of endurance and high intensity training on ICAM-1 and VCAM-1 levels and arterial pressure in obese and normal weight adolescents. Physician and Sportsmedicine (2016). | 10.1080/00913847.2016.1200442 | Setting | Intervention was not delivered in an eligible school setting. |
| 47 | Khammassi 2020 — Psycho-Physiological Responses to a 4-Month High-Intensity Interval Training-Centered Multidisciplinary Weight-Loss Intervention in Adolescents with Obesity. J Obes Metab Syndr (2020). | 10.7570/jomes20074 | Intervention | Intervention did not meet the prespecified HIIT eligibility criterion. |
| 48 | Khammassi 2018 — Impact of a 12-week high-intensity interval training without caloric restriction on body composition and lipid profile in sedentary healthy overweight/obese youth. J Exerc Rehabil (2018). | 10.12965/jer.1835124.562 | Population | Participant population was older than the prespecified upper age limit of 12 years. |
| 49 | Kojić 2026 — Enhancing Motor Fitness in Children Through Corrective Gymnastics in Physical Education. The Journal of School Health (2026). | https://doi.org/10.1111/josh.70148 | Intervention | Intervention did not meet the prespecified HIIT eligibility criterion. |
| 50 | Kvalo 2017 — Does increased physical activity in school affect children's executive function and aerobic fitness?. Scandinavian Journal of Medicine & Science in Sports (2017). | 10.1111/sms.12856 | Study design | Ineligible study design. |
| 51 | Liao 2024 — Effects of aquatic and land high-intensity interval trainings on selected bio- and physiological variables among obese adolescents. Frontiers in Endocrinology (2024). | 10.3389/fendo.2024.1381925 | Comparator | Comparator did not meet the prespecified eligibility criterion. |
| 52 | Lott 2017 — Executive control mediates the association between aerobic fitness and emotion regulation in preadolescent children. Journal of Pediatric Psychology (2017). | NR | Intervention | Intervention did not meet the prespecified HIIT eligibility criterion. |
| 53 | MacMahon 1987 — Physical and psychological effects of aerobic exercise in boys with learning disabilities. Journal of Developmental and Behavioral Pediatrics (1987). | https://dx.doi.org/10.1097/00004703-198710000-00006 | Population | Participant population did not meet the prespecified eligibility criterion. |
| 54 | Magnusson 2012 — Limited effects of a 2-year school-based physical activity intervention on body composition and cardiorespiratory fitness in 7-year-old children. Health Education Research (2012). | https://dx.doi.org/10.1093/her/cys049 | Intervention | Intervention did not meet the prespecified HIIT eligibility criterion. |
| 55 | Magnusson 2011 — Assessment of a two-year school-based physical activity intervention among 7-9-year-old children. International Journal of Behavioral Nutrition & Physical Activity (2011). | https://dx.doi.org/10.1186/1479-5868-8-138 | Intervention | Intervention did not meet the prespecified HIIT eligibility criterion. |
| 56 | Malden 2019 — The Daily Mile: teachers' perspectives of the barriers and facilitators to the delivery of a school-based physical activity intervention. BMJ Open (2019). | https://dx.doi.org/10.1136/bmjopen-2018-027169 | Study design | Ineligible study design. |
| 57 | Malik 2017 — Acute cardiorespiratory, perceptual and enjoyment responses tohigh-intensity interval exercise in adolescents. European Journal of Sport Science (2017). | 10.1080/17461391.2017.1364300 | Population | Participant population was older than the prespecified upper age limit of 12 years. |
| 58 | Malik 2018 — Perceptual Responses to High- and Moderate-Intensity Interval Exercise in Adolescents. Medicine and Science in Sports and Exercise (2018). | 10.1249/mss.0000000000001508 | Population | Participant population was older than the prespecified upper age limit of 12 years. |
| 59 | Malik 2019 — Perceptual and Cardiorespiratory Responses to High-Intensity Interval Exercise in Adolescents: Does Work Intensity Matter?. Journal of Sports Science and Medicine (2019). | WOS:000458299400001 | Study design | Study did not use an eligible controlled intervention design. |
| 60 | Malik 2021 — Influence of personality and self-efficacy on perceptual responses during high-intensity interval exercise in adolescents. Journal of Applied Sport Psychology (2021). | 10.1080/10413200.2020.1718798 | Intervention | Intervention did not meet the prespecified HIIT eligibility criterion. |
| 61 | Malina 2007 — Physical fitness of children and adolescents in the United States: status and secular change. Medicine & Sport Science (2007). | https://dx.doi.org/10.1159/000101076 | Study design | Study did not use an eligible controlled intervention design. |
| 62 | Manzano-Carrasco 2022 — Differences in body composition and physical fitness parameters among prepubertal and pubertal children engaged in extracurricular sports: The active health study. European Journal of Public Health (2022). | https://dx.doi.org/10.1093/eurpub/ckac075 | Study design | Cross-sectional study; no eligible intervention comparison. |
| 63 | Matta 2021 — Low sports participation is associated with withdrawn and depressed symptoms in Urban, school-age children. Journal of Affective Disorders (2021). | https://dx.doi.org/10.1016/j.jad.2020.11.076 | Study design | Ineligible study design. |
| 64 | Mazzoli 2019 — Associations of Class-Time Sitting, Stepping and Sit-to-Stand Transitions with Cognitive Functions and Brain Activity in Children. International Journal of Environmental Research and Public Health (2019). | https://doi.org/10.3390/ijerph16091482 | Study design | Ineligible study design. |
| 65 | McManus 2019 — The Impact of Different Exercise Intensities on Vasodilation and Shear Rate Patterns in Children. Pediatric Exercise Science (2019). | 10.1123/pes.2019-0003 | Intervention | Intervention did not meet the prespecified HIIT eligibility criterion. |
| 66 | McNarry 2019 — Effect of asthma and six-months high-intensity interval training on heart rate variability during exercise in adolescents. Journal of Sports Sciences (2019). | 10.1080/02640414.2019.1626115 | Comparator | Comparator did not meet the prespecified eligibility criterion. |
| 67 | Messiah 2018 — Sex differences in fitness outcomes among minority youth after participation in a park-based after-school program. Annals of Epidemiology (2018). | https://dx.doi.org/10.1016/j.annepidem.2018.03.020 | Intervention | Intervention did not meet the prespecified HIIT eligibility criterion. |
| 68 | Messler 2018 — Multimodal Therapy Involving High-Intensity Interval Training Improves the Physical Fitness, Motor Skills, Social Behavior, and Quality of Life of Boys With ADHD: A Randomized Controlled Study. Journal of Attention Disorders (2018). | 10.1177/1087054716636936 | Comparator | Comparator did not meet the prespecified eligibility criterion. |
| 69 | Mezcua-Hidalgo 2019 — 48-Hour Effects of Monitored Cooperative High-Intensity Interval Training on Adolescent Cognitive Functioning. Perceptual and Motor Skills (2019). | 10.1177/0031512518825197 | Intervention | Intervention did not meet the prespecified HIIT eligibility criterion. |
| 70 | Miguet 2020 — Effect of HIIT versus MICT on body composition and energy intake in dietary restrained and unrestrained adolescents with obesity. Applied Physiology Nutrition and Metabolism (2020). | 10.1139/apnm-2019-0160 | Intervention | Intervention did not meet the prespecified HIIT eligibility criterion. |
| 71 | Mitić 2024 — Enhancing Adolescent Physical Fitness and Well-Being: A School-Based High-Intensity Interval Training Program. J Funct Morphol Kinesiol (2024). | 10.3390/jfmk9040279 | Population | Participant population was older than the prespecified upper age limit of 12 years. |
| 72 | Morano 2020 — Physical Activity and Physical Competence in Overweight and Obese Children: An Intervention Study. International Journal of Environmental Research & Public Health [Electronic Resource] (2020). | https://dx.doi.org/10.3390/ijerph17176370 | Intervention | Intervention did not meet the prespecified HIIT eligibility criterion. |
| 73 | Morris 2018 — Food intake and appetite following school-based high-intensity interval training in 9-11-year-old children. Journal of Sports Sciences (2018). | 10.1080/02640414.2017.1302599 | Intervention | Intervention did not meet the prespecified HIIT eligibility criterion. |
| 74 | Murphy 2015 — The Feasibility of High-Intensity Interval Exercise in Obese Adolescents. Clinical Pediatrics (2015). | 10.1177/0009922814528038 | Study design | Study did not use an eligible controlled intervention design. |
| 75 | Oliveira 2018 — Mechanisms of blood pressure control following acute exercise in adolescents: Effects of exercise intensity on haemodynamics and baroreflex sensitivity. Experimental Physiology (2018). | 10.1113/ep086999 | Intervention | Intervention did not meet the prespecified HIIT eligibility criterion. |
| 76 | Osiński 2017 — Physical activity in the therapy of overweight and obesity in children and adolescents. Needs and recommendations for intervention programs. Dev Period Med (2017). | 10.34763/devperiodmed.20172103.224234 | Study design | Review article; not an eligible primary intervention study. |
| 77 | Pastor 2021 — Acute physical exercise intensity, cognitive inhibition and psychological well-being in adolescent physical education students. Current Psychology (2021). | 10.1007/s12144-019-00454-z | Intervention | Intervention did not meet the prespecified HIIT eligibility criterion. |
| 78 | Racil 2023 — High-Intensity Interval Training in Female Adolescents with Moderate or Severe Obesity. Children-Basel (2023). | 10.3390/children10091495 | Population | Participant population was older than the prespecified upper age limit of 12 years. |
| 79 | Segovia 2020 — Effect of a game-based high intensity interval training program on body composition in primary education: comparison of the Sport Education model and traditional methodology. Journal of Physical Education and Sport (2020). | https://doi.org/10.7752/jpes.2020.02113 | Study design | Uncontrolled single-group pre–post quasi-experimental design. |
| 80 | Sepúlveda-Flores 2025 — Comparación de los efectos de dos protocolos de entrenamiento (HIIT vs. MICT) sobre la composición corporal y los marcadores metabólicos en adolescentes con obesidad. Salud Uninorte (2025). | https://doi.org/10.14482/sun.41.01.158.245 | Language | Report was not published in English. |
| 81 | Sharp 2020 — Identifying facilitators and barriers for adolescents participating in a school-based HIIT intervention: the eXercise for asthma with commando Joe's® (X4ACJ) programme. BMC Public Health (2020). | 10.1186/s12889-020-08740-3 | Population | Participant population was older than the prespecified upper age limit of 12 years. |
| 82 | Simonsen 2025 — Intensity and enjoyment of play-based HIIT in children and adolescents with obesity. International Journal of Sports Medicine (2025). | 10.1055/a-2456-4054 | Population | Participant population was older than the prespecified upper age limit of 12 years. |
| 83 | Tottori 2019 — Effects of High Intensity Interval Training on Executive Function in Children Aged 8-12 Years. Int J Environ Res Public Health (2019). | 10.3390/ijerph16214127 | Study design | Study did not use an eligible controlled intervention design. |
| 84 | Valdivieso-González 2025 — Changes in cortical brain activity after active break in preschoolers: MOVI-HIIT study. Frontiers in Sports and Active Living (2025). | 10.3389/fspor.2025.1529288 | Population | Participant population was younger than the prespecified lower age limit of 5 years. |
| 85 | vanBiljon 2018 — Do Short-Term Exercise Interventions Improve Cardiometabolic Risk Factors in Children?. Journal of Pediatrics (2018). | 10.1016/j.jpeds.2018.07.067 | Study design | Study did not use an eligible controlled intervention design. |
| 86 | Wang 2024 — Comparative study on the function of high-intensity intermittent exercise and continuous aerobic exercise on obese children. Medicina Balear (2024). | 10.3306/ajhs.2024.39.05.40 | Setting | Intervention was not delivered in an eligible school setting. |
| 87 | Wenping 2024 — Effects of High-Intensity Interval Training on Cardiopulmonary Fitness and Blood Pressure of Obese Adolescent Girls in China. Journal of Electrical Systems (2024). | 3092061870 | Population | Participant population was older than the prespecified upper age limit of 12 years. |
| 88 | Zhang 2022 — Effects of Acute High-Intensity Interval Exercise and High-Intensity Continuous Exercise on Inhibitory Function of Overweight and Obese Children. International Journal of Environmental Research and Public Health (2022). | https://doi.org/10.3390/ijerph191610401 | Intervention | Intervention did not meet the prespecified HIIT eligibility criterion. |
