# Supplementary material for: School-based high-intensity interval training for children: a focused systematic review of implementation and school-relevant outcomes
Source: Front Pediatr. 2026 Sep 28;14:1952928. doi: 10.3389/fped.2026.1952928 (PMC13655682; doi:10.3389/fped.2026.1952928)
Supplement: Supplementary file 3 [file Supplementaryfile3.docx]

**Supplemental File 3**

**Risk-of-Bias Assessment (RoB 2) for Included Studies**

*School-Based High-Intensity Interval Training for Children and Adolescents:*A Focused Systematic Review of Implementation and School-Relevant Outcomes

**Contents**

| **Table title** | **Page** |
| --- | --- |
| Table 1. Risk-of-bias assessment for Engel et al. (13) | 2 |
| Table 2. Risk-of-bias assessment for Ketelhut et al. (14) | 4 |
| Table 3. Risk-of-bias assessment for Ketelhut et al. (17) | 6 |
| Table 4. Risk-of-bias assessment for Liu et al. (18) | 8 |
| Table 5. Risk-of-bias assessment for Mucci et al. (15) | 10 |
| Table 6. Risk-of-bias assessment for Ricci et al. (16) | 12 |

# **Table 1. Risk-of-bias assessment for Engel et al. (13)**

|  | **Domain** | **Signalling question** | **Response** |
| --- | --- | --- | --- |
| **Engel et al. (13)** | **Bias arising from the randomization process** | 1.1 Was the allocation sequence random? | PY |
|  |  | 1.2 Was the allocation sequence concealed until participants were enrolled and assigned to interventions? | NI |
|  |  | 1.3 Did baseline differences between intervention groups suggest a problem with the randomization process? | PN |
|  |  | **Risk of bias judgement** | **Some concerns** |
|  | **Bias due to deviations from intended interventions** | 2.1.Were participants aware of their assigned intervention during the trial? | Y |
|  |  | 2.2.Were carers and people delivering the interventions aware of participants' assigned intervention during the trial? | Y |
|  |  | 2.3. If Y/PY/NI to 2.1 or 2.2: Were there deviations from the intended intervention that arose because of the experimental context? | PY |
|  |  | 2.4 If Y/PY to 2.3: Were these deviations likely to have affected the outcome? | PY |
|  |  | 2.5. If Y/PY/NI to 2.4: Were these deviations from intended intervention balanced between groups? | NI |
|  |  | 2.6 Was an appropriate analysis used to estimate the effect of assignment to intervention? | PN |
|  |  | 2.7 If N/PN/NI to 2.6: Was there potential for a substantial impact (on the result) of the failure to analyse participants in the group to which they were randomized? | PN |
|  |  | **Risk of bias judgement** | **Some concerns** |
|  | **Bias due to missing outcome data** | 3.1 Were data for this outcome available for all, or nearly all, participants randomized? | PN |
|  |  | 3.2 If N/PN/NI to 3.1: Is there evidence that result was not biased by missing outcome data? | PN |
|  |  | 3.3 If N/PN to 3.2: Could missingness in the outcome depend on its true value? | PY |
|  |  | 3.4 If Y/PY/NI to 3.3: Is it likely that missingness in the outcome depended on its true value? | NI |
|  |  | **Risk of bias judgement** | **Some concerns** |
|  | **Bias in measurement of the outcome** | 4.1 Was the method of measuring the outcome inappropriate? | N |
|  |  | 4.2 Could measurement or ascertainment of the outcome have differed between intervention groups? | PN |
|  |  | 4.3 Were outcome assessors aware of the intervention received by study participants? | PY |
|  |  | 4.4 If Y/PY/NI to 4.3: Could assessment of the outcome have been influenced by knowledge of intervention received? | PY |
|  |  | 4.5 If Y/PY/NI to 4.4: Is it likely that assessment of the outcome was influenced by knowledge of intervention received? | PN |
|  |  | **Risk of bias judgement** | **Some concerns** |
|  | **Bias in selection of the reported result** | 5.1 Were the data that produced this result analysed in accordance with a pre-specified analysis plan that was finalized before unblinded outcome data were available for analysis? | NI |
|  |  | 5.2 ... multiple eligible outcome measurements (e.g. scales, definitions, time points) within the outcome domain? | PY |
|  |  | 5.3 ... multiple eligible analyses of the data? | PY |
|  |  | **Risk of bias judgement** | **Some concerns** |
|  | **Overall bias** | **Risk of bias judgement** | **Some concerns** |

# **Table 2. Risk-of-bias assessment for Ketelhut et al. (14)**

|  | **Domain** | **Signalling question** | **Response** |
| --- | --- | --- | --- |
| **Ketelhut et al. (14)** | **Bias arising from the randomization process** | 1.1 Was the allocation sequence random? | Y |
|  |  | 1.2 Was the allocation sequence concealed until participants were enrolled and assigned to interventions? | NI |
|  |  | 1.3 Did baseline differences between intervention groups suggest a problem with the randomization process? | PN |
|  |  | **Risk of bias judgement** | **Some concerns** |
|  | **Bias due to deviations from intended interventions** | 2.1.Were participants aware of their assigned intervention during the trial? | Y |
|  |  | 2.2.Were carers and people delivering the interventions aware of participants' assigned intervention during the trial? | Y |
|  |  | 2.3. If Y/PY/NI to 2.1 or 2.2: Were there deviations from the intended intervention that arose because of the experimental context? | Y |
|  |  | 2.4 If Y/PY to 2.3: Were these deviations likely to have affected the outcome? | PN |
|  |  | 2.5. If Y/PY/NI to 2.4: Were these deviations from intended intervention balanced between groups? | NA |
|  |  | 2.6 Was an appropriate analysis used to estimate the effect of assignment to intervention? | N |
|  |  | 2.7 If N/PN/NI to 2.6: Was there potential for a substantial impact (on the result) of the failure to analyse participants in the group to which they were randomized? | Y |
|  |  | **Risk of bias judgement** | **High** |
|  | **Bias due to missing outcome data** | 3.1 Were data for this outcome available for all, or nearly all, participants randomized? | N |
|  |  | 3.2 If N/PN/NI to 3.1: Is there evidence that result was not biased by missing outcome data? | N |
|  |  | 3.3 If N/PN to 3.2: Could missingness in the outcome depend on its true value? | PY |
|  |  | 3.4 If Y/PY/NI to 3.3: Is it likely that missingness in the outcome depended on its true value? | PN |
|  |  | **Risk of bias judgement** | **Some concerns** |
|  | **Bias in measurement of the outcome** | 4.1 Was the method of measuring the outcome inappropriate? | N |
|  |  | 4.2 Could measurement or ascertainment of the outcome have differed between intervention groups? | PN |
|  |  | 4.3 Were outcome assessors aware of the intervention received by study participants? | NI |
|  |  | 4.4 If Y/PY/NI to 4.3: Could assessment of the outcome have been influenced by knowledge of intervention received? | PN |
|  |  | 4.5 If Y/PY/NI to 4.4: Is it likely that assessment of the outcome was influenced by knowledge of intervention received? | NA |
|  |  | **Risk of bias judgement** | **Low** |
|  | **Bias in selection of the reported result** | 5.1 Were the data that produced this result analysed in accordance with a pre-specified analysis plan that was finalized before unblinded outcome data were available for analysis? | NI |
|  |  | 5.2 ... multiple eligible outcome measurements (e.g. scales, definitions, time points) within the outcome domain? | PY |
|  |  | 5.3 ... multiple eligible analyses of the data? | PY |
|  |  | **Risk of bias judgement** | **Some concerns** |
|  | **Overall bias** | **Risk of bias judgement** | **High** |

# **Table 3. Risk-of-bias assessment for Ketelhut et al. (17)**

|  | **Domain** | **Signalling question** | **Response** |
| --- | --- | --- | --- |
| **Ketelhut et al. (17)** | **Bias arising from the randomization process** | 1.1 Was the allocation sequence random? | Y |
|  |  | 1.2 Was the allocation sequence concealed until participants were enrolled and assigned to interventions? | NI |
|  |  | 1.3 Did baseline differences between intervention groups suggest a problem with the randomization process? | PY |
|  |  | **Risk of bias judgement** | **High** |
|  | **Bias due to deviations from intended interventions** | 2.1.Were participants aware of their assigned intervention during the trial? | Y |
|  |  | 2.2.Were carers and people delivering the interventions aware of participants' assigned intervention during the trial? | Y |
|  |  | 2.3. If Y/PY/NI to 2.1 or 2.2: Were there deviations from the intended intervention that arose because of the experimental context? | PN |
|  |  | 2.4 If Y/PY to 2.3: Were these deviations likely to have affected the outcome? | NA |
|  |  | 2.5. If Y/PY/NI to 2.4: Were these deviations from intended intervention balanced between groups? | NA |
|  |  | 2.6 Was an appropriate analysis used to estimate the effect of assignment to intervention? | PY |
|  |  | 2.7 If N/PN/NI to 2.6: Was there potential for a substantial impact (on the result) of the failure to analyse participants in the group to which they were randomized? | NA |
|  |  | **Risk of bias judgement** | **Low** |
|  | **Bias due to missing outcome data** | 3.1 Were data for this outcome available for all, or nearly all, participants randomized? | PY |
|  |  | 3.2 If N/PN/NI to 3.1: Is there evidence that result was not biased by missing outcome data? | NA |
|  |  | 3.3 If N/PN to 3.2: Could missingness in the outcome depend on its true value? | NA |
|  |  | 3.4 If Y/PY/NI to 3.3: Is it likely that missingness in the outcome depended on its true value? | NA |
|  |  | **Risk of bias judgement** | **Low** |
|  | **Bias in measurement of the outcome** | 4.1 Was the method of measuring the outcome inappropriate? | N |
|  |  | 4.2 Could measurement or ascertainment of the outcome have differed between intervention groups? | PN |
|  |  | 4.3 Were outcome assessors aware of the intervention received by study participants? | NI |
|  |  | 4.4 If Y/PY/NI to 4.3: Could assessment of the outcome have been influenced by knowledge of intervention received? | PY |
|  |  | 4.5 If Y/PY/NI to 4.4: Is it likely that assessment of the outcome was influenced by knowledge of intervention received? | PN |
|  |  | **Risk of bias judgement** | **Some concerns** |
|  | **Bias in selection of the reported result** | 5.1 Were the data that produced this result analysed in accordance with a pre-specified analysis plan that was finalized before unblinded outcome data were available for analysis? | NI |
|  |  | 5.2 ... multiple eligible outcome measurements (e.g. scales, definitions, time points) within the outcome domain? | PY |
|  |  | 5.3 ... multiple eligible analyses of the data? | PY |
|  |  | **Risk of bias judgement** | **Some concerns** |
|  | **Overall bias** | **Risk of bias judgement** | **High** |

# **Table 4. Risk-of-bias assessment for Liu et al. (18)**

|  | **Domain** | **Signalling question** | **Response** |
| --- | --- | --- | --- |
| **Liu et al. (18)** | **Bias arising from the randomization process** | 1.1 Was the allocation sequence random? | Y |
|  |  | 1.2 Was the allocation sequence concealed until participants were enrolled and assigned to interventions? | NI |
|  |  | 1.3 Did baseline differences between intervention groups suggest a problem with the randomization process? | NI |
|  |  | **Risk of bias judgement** | **Some concerns** |
|  | **Bias due to deviations from intended interventions** | 2.1.Were participants aware of their assigned intervention during the trial? | Y |
|  |  | 2.2.Were carers and people delivering the interventions aware of participants' assigned intervention during the trial? | Y |
|  |  | 2.3. If Y/PY/NI to 2.1 or 2.2: Were there deviations from the intended intervention that arose because of the experimental context? | NI |
|  |  | 2.4 If Y/PY to 2.3: Were these deviations likely to have affected the outcome? | NA |
|  |  | 2.5. If Y/PY/NI to 2.4: Were these deviations from intended intervention balanced between groups? | NA |
|  |  | 2.6 Was an appropriate analysis used to estimate the effect of assignment to intervention? | N |
|  |  | 2.7 If N/PN/NI to 2.6: Was there potential for a substantial impact (on the result) of the failure to analyse participants in the group to which they were randomized? | PY |
|  |  | **Risk of bias judgement** | **High** |
|  | **Bias due to missing outcome data** | 3.1 Were data for this outcome available for all, or nearly all, participants randomized? | PN |
|  |  | 3.2 If N/PN/NI to 3.1: Is there evidence that result was not biased by missing outcome data? | N |
|  |  | 3.3 If N/PN to 3.2: Could missingness in the outcome depend on its true value? | PY |
|  |  | 3.4 If Y/PY/NI to 3.3: Is it likely that missingness in the outcome depended on its true value? | NI |
|  |  | **Risk of bias judgement** | **Some concerns** |
|  | **Bias in measurement of the outcome** | 4.1 Was the method of measuring the outcome inappropriate? | N |
|  |  | 4.2 Could measurement or ascertainment of the outcome have differed between intervention groups? | N |
|  |  | 4.3 Were outcome assessors aware of the intervention received by study participants? | NI |
|  |  | 4.4 If Y/PY/NI to 4.3: Could assessment of the outcome have been influenced by knowledge of intervention received? | N |
|  |  | 4.5 If Y/PY/NI to 4.4: Is it likely that assessment of the outcome was influenced by knowledge of intervention received? | NA |
|  |  | **Risk of bias judgement** | **Low** |
|  | **Bias in selection of the reported result** | 5.1 Were the data that produced this result analysed in accordance with a pre-specified analysis plan that was finalized before unblinded outcome data were available for analysis? | NI |
|  |  | 5.2 ... multiple eligible outcome measurements (e.g. scales, definitions, time points) within the outcome domain? | Y |
|  |  | 5.3 ... multiple eligible analyses of the data? | PY |
|  |  | **Risk of bias judgement** | **Some concerns** |
|  | **Overall bias** | **Risk of bias judgement** | **High** |

# **Table 5. Risk-of-bias assessment for Mucci et al. (15)**

|  | **Domain** | **Signalling question** | **Response** |
| --- | --- | --- | --- |
| **Mucci et al. (15)** | **Bias arising from the randomization process** | 1.1 Was the allocation sequence random? | PY |
|  |  | 1.2 Was the allocation sequence concealed until participants were enrolled and assigned to interventions? | NI |
|  |  | 1.3 Did baseline differences between intervention groups suggest a problem with the randomization process? | N |
|  |  | **Risk of bias judgement** | **Some concerns** |
|  | **Bias due to deviations from intended interventions** | 2.1.Were participants aware of their assigned intervention during the trial? | Y |
|  |  | 2.2.Were carers and people delivering the interventions aware of participants' assigned intervention during the trial? | Y |
|  |  | 2.3. If Y/PY/NI to 2.1 or 2.2: Were there deviations from the intended intervention that arose because of the experimental context? | N |
|  |  | 2.4 If Y/PY to 2.3: Were these deviations likely to have affected the outcome? | NA |
|  |  | 2.5. If Y/PY/NI to 2.4: Were these deviations from intended intervention balanced between groups? | NA |
|  |  | 2.6 Was an appropriate analysis used to estimate the effect of assignment to intervention? | PY |
|  |  | 2.7 If N/PN/NI to 2.6: Was there potential for a substantial impact (on the result) of the failure to analyse participants in the group to which they were randomized? | NA |
|  |  | **Risk of bias judgement** | **Low** |
|  | **Bias due to missing outcome data** | 3.1 Were data for this outcome available for all, or nearly all, participants randomized? | N |
|  |  | 3.2 If N/PN/NI to 3.1: Is there evidence that result was not biased by missing outcome data? | N |
|  |  | 3.3 If N/PN to 3.2: Could missingness in the outcome depend on its true value? | PY |
|  |  | 3.4 If Y/PY/NI to 3.3: Is it likely that missingness in the outcome depended on its true value? | NI |
|  |  | **Risk of bias judgement** | **Some concerns** |
|  | **Bias in measurement of the outcome** | 4.1 Was the method of measuring the outcome inappropriate? | N |
|  |  | 4.2 Could measurement or ascertainment of the outcome have differed between intervention groups? | N |
|  |  | 4.3 Were outcome assessors aware of the intervention received by study participants? | NI |
|  |  | 4.4 If Y/PY/NI to 4.3: Could assessment of the outcome have been influenced by knowledge of intervention received? | PN |
|  |  | 4.5 If Y/PY/NI to 4.4: Is it likely that assessment of the outcome was influenced by knowledge of intervention received? | NA |
|  |  | **Risk of bias judgement** | **Low** |
|  | **Bias in selection of the reported result** | 5.1 Were the data that produced this result analysed in accordance with a pre-specified analysis plan that was finalized before unblinded outcome data were available for analysis? | NI |
|  |  | 5.2 ... multiple eligible outcome measurements (e.g. scales, definitions, time points) within the outcome domain? | N |
|  |  | 5.3 ... multiple eligible analyses of the data? | PY |
|  |  | **Risk of bias judgement** | **Some concerns** |
|  | **Overall bias** | **Risk of bias judgement** | **Some concerns** |

# **Table 6. Risk-of-bias assessment for Ricci et al. (16)**

|  | **Domain** | **Signalling question** | **Response** |
| --- | --- | --- | --- |
| **Ricci et al. (16)** | **Bias arising from the randomization process** | 1.1 Was the allocation sequence random? | PY |
|  |  | 1.2 Was the allocation sequence concealed until participants were enrolled and assigned to interventions? | NI |
|  |  | 1.3 Did baseline differences between intervention groups suggest a problem with the randomization process? | PN |
|  |  | **Risk of bias judgement** | **Some concerns** |
|  | **Bias due to deviations from intended interventions** | 2.1.Were participants aware of their assigned intervention during the trial? | Y |
|  |  | 2.2.Were carers and people delivering the interventions aware of participants' assigned intervention during the trial? | Y |
|  |  | 2.3. If Y/PY/NI to 2.1 or 2.2: Were there deviations from the intended intervention that arose because of the experimental context? | N |
|  |  | 2.4 If Y/PY to 2.3: Were these deviations likely to have affected the outcome? | NA |
|  |  | 2.5. If Y/PY/NI to 2.4: Were these deviations from intended intervention balanced between groups? | NA |
|  |  | 2.6 Was an appropriate analysis used to estimate the effect of assignment to intervention? | Y |
|  |  | 2.7 If N/PN/NI to 2.6: Was there potential for a substantial impact (on the result) of the failure to analyse participants in the group to which they were randomized? | NA |
|  |  | **Risk of bias judgement** | **Low** |
|  | **Bias due to missing outcome data** | 3.1 Were data for this outcome available for all, or nearly all, participants randomized? | N |
|  |  | 3.2 If N/PN/NI to 3.1: Is there evidence that result was not biased by missing outcome data? | PN |
|  |  | 3.3 If N/PN to 3.2: Could missingness in the outcome depend on its true value? | NI |
|  |  | 3.4 If Y/PY/NI to 3.3: Is it likely that missingness in the outcome depended on its true value? | NI |
|  |  | **Risk of bias judgement** | **Some concerns** |
|  | **Bias in measurement of the outcome** | 4.1 Was the method of measuring the outcome inappropriate? | N |
|  |  | 4.2 Could measurement or ascertainment of the outcome have differed between intervention groups? | N |
|  |  | 4.3 Were outcome assessors aware of the intervention received by study participants? | PY |
|  |  | 4.4 If Y/PY/NI to 4.3: Could assessment of the outcome have been influenced by knowledge of intervention received? | PY |
|  |  | 4.5 If Y/PY/NI to 4.4: Is it likely that assessment of the outcome was influenced by knowledge of intervention received? | PN |
|  |  | **Risk of bias judgement** | **Low** |
|  | **Bias in selection of the reported result** | 5.1 Were the data that produced this result analysed in accordance with a pre-specified analysis plan that was finalized before unblinded outcome data were available for analysis? | NI |
|  |  | 5.2 ... multiple eligible outcome measurements (e.g. scales, definitions, time points) within the outcome domain? | N |
|  |  | 5.3 ... multiple eligible analyses of the data? | PY |
|  |  | **Risk of bias judgement** | **Some concerns** |
|  | **Overall bias** | **Risk of bias judgement** | **Some concerns** |
